# A cross-sectional study on advance care planning documentation attitudes during national advance care planning week in a South-East Asian country

**DOI:** 10.1186/s12904-024-01505-4

**Published:** 2024-10-17

**Authors:** Chen Ee Low, Sounak Rana, Chun En Yau, Sheryl Yen Pin Tan, Jing Ni Ng, Noreen Chan, Mervyn Jun Rui Lim

**Affiliations:** 1 Yong Loo Lin School of Medicine, National University of Singapore, Singapore, Republic of Singapore; 2 Division of Palliative Care, National University Cancer Institute, Singapore, Republic of Singapore; 3 Division of Neurosurgery, University Surgical Centre, National University Hospital, Level 8, National University Health Systems Tower Block, 1E Kent Ridge Rd, Singapore, 119228 Republic of Singapore

**Keywords:** Palliative care, Advance care planning, Exhibition, Singapore, Covid-19

## Abstract

**Background:**

Through advocacy initiatives such as roadshows during “National ACP Week”, the Agency for Integrated Care (AIC) had increased advance care planning (ACP) engagement since 2011. Project Happy Apples (PHA), a community initiative project led by medical students from the National University of Singapore, also conducted a public exhibition to raise ACP awareness during this period. This study aimed to investigate and identify predictors of attitudes towards ACP documentation among ‘ACP Week’ respondents which may be used to formulate strategies to increase ACP documentation in Singapore.

**Methods:**

A cross-sectional study on ACP documentation attitudes of 262 respondents during local roadshows were conducted. Multiple logistic regression models were built to investigate the associations between demographic variables and attitudes toward ACP documentation.

**Results:**

The mean age was 43.5 years (SD = 17.4), 79 (30.15%) were males and 49 (18.7%) were healthcare professionals (HCP). 117 (44.66%) respondents had prior experience with serious illness and 116 (44.27%) had heard of ACP. Age was a significant predictor of readiness to sign official papers naming nominated healthcare spokesperson (NHS) (OR = 1.04, 95%CI: 1.02–1.07). Experience with serious illness was a significant predictor of readiness to discuss end-of-life (EOL) care with healthcare professionals (HCP) (OR = 3.65, 95%CI: 1.36–11.61). Being female was a significant predictor for readiness to speak to their nominated healthcare spokesperson about EOL care (OR = 7.33, 95%CI: 2.06–46.73). Subgroup analyses revealed that those aged 20–39 were less likely to speak to their healthcare professional about or sign official papers regarding EOL care. We also found that being a healthcare professional does not necessitate better or worse attitudes.

**Conclusion:**

Advocacy programs tailored to targeting respondents of different age groups and prior experience with serious illness may improve the efficacy of advocacy efforts.

**Supplementary Information:**

The online version contains supplementary material available at 10.1186/s12904-024-01505-4.

## Introduction

Quality end-of-life (EOL) care emerged as an important public health issue in developed countries in the last 30 years [1–3]. Palliative care is an approach that improves the quality of life of patients and their families who are facing problems associated with a life-threatening illness. It prevents and relieves suffering through the early identification, correct assessment and treatment of pain and other problems, whether physical, psychosocial, or spiritual [4]. It had been demonstrated to improve the quality of life for patients and their loved ones [5, 6].

As defined by Sudore et al. [7] (2017), advance care planning (ACP) is a process that supports adults at any age or stage of health in understanding and sharing their personal values, life goals, and preferences regarding future medical care [7]. ACP has been shown to be effective in improving patient outcomes, providing better EOL care, reducing stress in surviving relatives, and higher patient-family satisfaction [8]. Having early ACP conversations may offer family members and loved ones sufficient time to explore proper EOL care that addresses not just the medical decisions, but also the emotional, relational, social, legal, and financial aspects [9] that must be taken into consideration when administering EOL care. However, members of the public in Asian communities often consider such conversations taboo and avoid them [10–12].

Singapore’s Ministry of Health commissioned the Agency of Integrated Care to coordinate health initiatives for older adults [13]. The Living Matters ACP model [14], adapted from the Respecting Choices model [15] from the United States, was formally implemented through the Agency of Integrated Care in 2011–201,215. The model sought to promote open conversations between families, and healthcare providers about future care preferences. As part of their advocacy model, ‘National ACP Week’ [16] occurred annually from the 9th to the 15th of May in Singapore where various ACP service providers would be invited to jointly organize events at a roadshow also known as ‘ACP Roadshow’. These initiatives were effective in increasing ACP awareness locally, as reported by the rising uptake rate of ACP, totalling 26,627 ACP documents published from 2013 to 2021 [17]. However, with a population of more than five million in Singapore, awareness of the ACP conversation among the general population remained low [18].

Project Happy Apples [19] was a local community-involvement project led by students from the National University of Singapore, Yong Loo Lin School of Medicine (NUS YLLSoM) which aimed to raise public awareness on palliative care and ACP. Project Happy Apples hosted annual exhibitions in major malls to facilitate end-of-life care conversations and raise awareness of palliative care and ACP. This was achieved through interactive exhibits, information boards and active facilitation by invited speakers and medical students. Both Project Happy Apples’ annual exhibition and the ACP Roadshow [20] were held in conjunction with National ACP Week 2022 [18].

While previous studies have been conducted examining ACP awareness and attitudes in the Singapore public [21], there was a lack of literature focusing on assessing the readiness of this population to engage with the ACP documentation process. In addition, efforts to promote ACP uptake in 2020 and 2021 were complicated by COVID-19 [22]. COVID-19 was a pandemic that had multifaceted effects on both health policies and the healthcare systems of various countries [23]. National ACP Week took place during the pandemic in Singapore. The pandemic may have shaped the perspectives of the public on EOL care, which warranted an investigation. Therefore, we aimed to investigate the associations between the respondent characteristics of the Singaporean public and their readiness to engage with the ACP documentation process during the Agency of Integrated Care National ACP Week in 2022.

## Methodology

### Study-design

This was a cross-sectional study of 262 survey respondents during National ACP Week 2022. There were 54 respondents at the Agency of Integrated Care Roadshow and 208 respondents at Project Happy Apples’ exhibition. A standardized anonymous questionnaire on ACP Documentation Attitudes among members of the public was developed in collaboration with the Agency of Integrated Care and in consultation with a panel of multi-institutional registered palliative care specialists practising in Singapore. The questionnaire was modified to yield two versions for the ACP Roadshow, and the Project Happy Apples exhibition respectively (Supplementary Material 1–2). The inclusion criteria for this study were: (1) Singaporean citizen or permanent resident; (2) At least 18 years of age; (3) Proficient in English; (4) Able to provide informed consent.

A convenience sampling method was chosen to facilitate ease of administration in a roadshow and exhibition setting. The questionnaires were administered to roadshow and exhibition attendees by onsite facilitators. All facilitators received standardised interviewer training. Verbal consent was elicited from each participant, and a participant information sheet was provided. At both events, the questionnaires were administered via a Quick Response (QR) code and all survey responses were recorded on a secure online platform. Institutional ethics approval was obtained for both the Agency of Integrated Care Roadshow (National Healthcare Group Domain Specific Review Board (NHG DSRB) Ref: 2022/00282) and the PHA exhibition (NHG DSRB Ref: 2022/00462) prior to the conduct of the study. NHG DSRB has approved a waiver of written informed consent as the study held no more than minimal risk for the study’s participants.

### Questionnaire

The questionnaire was created with reference to the 4-item version of the ACP Engagement Survey [24], chosen to facilitate its ease of administration in an exhibition setting. The 4-item survey was an abbreviated version of the 82-item ACP Engagement Survey which focused on four behaviour change constructs within four ACP domains. The constructs were knowledge, contemplation, self-efficacy, and readiness. The domains are surrogate decision makers, values and quality of life, leeway in surrogate decision making, and asking doctors questions. The four items are as follows- “Ready to sign official papers naming a person or group of people to be your spokesperson or to make decisions for you”, “Ready to speak with your Nominated Healthcare Spokesperson about the kind of medical care you would want if you were near the end of life”, “Ready to speak with your Healthcare Professional about the kind of medical care you would want if you were near the end of life”, “Ready to sign official papers putting your wishes in writing about the kind of medical care you would want if you were near the end of life”. The following variables were collected: gender, age, race, religion, nationality, marital status, “Are you a healthcare professional?”, “Prior experience with a serious illness?”, “Have you thought about EOL care before?”, “Have you heard of ACP before?”, “Where have you heard of ACP?” and the 4 items representing ACP documentation attitudes. The ACP Engagement Survey has an overall Cronbach’s α of 0.84 [24].

The two sets of questionnaires, modified for application in the ACP Roadshow and PHA exhibition respectively, could be found in “Supplementary Materials”. No identifiable data was collected via the survey.

### Statistical analysis

Respondent demographics were summarised in Table 1. Categorical variables such as gender, religion, and whether someone has prior experience with serious illness, was a healthcare professional or has heard of ACP were reported as the number of responses and percentage. Continuous variables, such as age and the four markers for attitude towards ACP documentation were reported as mean and standard deviation (SD). Each of the four markers was ranked on a Likert scale from one to five, with one to three classified as negative ACP documentation attitudes, and four to five classified as positive ACP documentation. The markers were then analysed as a binomial categorical variable after classification. Multiple logistic regression models were used to investigate the associations between respondent demographics and the four attitudes towards ACP documentation. The confounders adjusted for were gender, age, religion, having heard of ACP, having prior experience with serious illness and being an healthcare professional. Effect sizes for the association were reported as odds ratio with 95% confidence intervals and *p*-values. A *p*-value of less than 0.05 was considered as statistically significant. All analyses were conducted with RStudio (Version 4.1.0).

**Table 1:** General characteristics of ACP Roadshow respondents (*n* = 262)

Characteristics	Respondents, *n* = 262(%)
Gender	
Male	79 (30.15)
Age	43.5 ± 17.4
Religion	
Buddhism	91 (34.70)
Christianity/Catholicism	80 (30.53)
Others	21 (8.00)
No religion	70 (26.70)
Is a Healthcare professional?	
No	213 (81.30)
Yes	49 (18.70)
Had prior experience with serious illness?	
No	117 (44.66)
Yes	145 (55.34)
Heard of advance care planning?	
No	116 (44.27)
Yes	146 (55.73)

## Results

262 respondents responded to the survey, of which there were 79 (30.15%) males and 183 (69.85%) females. The respondents’ mean age was 43.5 ± 17.4 years. Respondents comprised 91 Buddhists (34.7%), 80 Christians (30.5%), 70 non-religious (26.7%) and 21 from other religions such as Hinduism, Islam, Sikhism, Confucianism and Atheists (8.0%). There were 49 healthcare professionals (18.70%) and 213 non-healthcare professionals (81.30%). 145 respondents (55.34%) had prior experience with serious illness and 146 respondents (55.73%) had heard of advance care planning (Table 1).

Table 2 presented the ACP documentation attitudes of the respondents. Among the 262 respondents, the mean scores were as follows: Readiness to sign official papers naming a nominated healthcare spokesperson (2.31, SD = 1.24), readiness to speak with their nominated healthcare spokesperson about their preferred medical care at the end-of-life (2.12, SD = 1.18), readiness to speak with their healthcare professional about their preferred medical care at the end of life (2.00, SD = 1.05) and readiness to sign official papers listing their wishes in writing about their preferred medical care at the end-of-life (2.08, SD = 1.08). Across the four markers of ACP documentation attitudes, majority of the respondents had negative attitudes.

**Table 2:** Four markers of ACP Documentation attitudes of the respondents

ACP Documentation Attitudes	Scores from each marker		Attitude	
	Mean	Standard Deviation	Positive attitudes (*n*, %)	Negative attitudes (*n*, %)
Ready to sign official papers naming a person or group of people to be your spokesperson or to make decisions for you	2.31	1.24	40 (15.3)	222 (84.7)
Ready to speak with your Nominated Healthcare Spokesperson about the kind of medical care you would want if you were near the end of life	2.12	1.18	29 (11.1)	233 (88.9)
Ready to speak with your Healthcare Professional about the kind of medical care you would want if you were near the end of life	2.00	1.05	23 (8.8)	239 (91.2)
Ready to sign official papers putting your wishes in writing about the kind of medical care you would want if you were near the end of life	2.08	1.08	26 (9.9)	236 (90.1)

On adjusted multivariate logistic regression (Table 3), prior experience with serious illness was significantly correlated with readiness to speak with a healthcare professional about preferred EOL medical care (OR = 3.65, 95%CI: 1.36–11.61). Age was significantly associated with readiness to name a nominated healthcare spokesperson (OR = 1.04, 95%CI: 1.02–1.07). Being female was significantly associated with readiness to speak with a nominated healthcare spokesperson about preferred EOL care (OR = 7.33, 95%CI: 2.06–46.73). Sensitivity analyses was performed investigating healthcare professionals and the general public as individual populations (Supplementary Tables 1 and 2). Despite the small sample size, the trend in results remain largely similar in the general population and insignificant for healthcare professionals. For healthcare professionals, age, religion, gender, serious illness experience, and having heard of ACP were not significantly associated with any ACP documentation attitudes (Supplementary Table 1). Sensitivity analyses by age group as a categorical variable were also performed (Supplementary Table 3). Being aged 20–39 significantly associated with being less ready to speak with healthcare professionals regarding EOL care (OR = 0.24, 95%CI: 0.05–0.91) and less ready to sign official papers regarding EOL care (OR = 0.28, 95%CI: 0.07–0.94).

**Table 3:** Logistic regressions of the association between the four markers of ACP Documentation Attitudes and general characteristics Overall

	Ready to sign official papers naming a nominated healthcare spokesperson			Ready to speak with your nominated healthcare spokesperson about your preferred EOL medical care			Ready to speak with your healthcare professional about your preferred EOL medical care			Ready to sign official papers putting your wishes in writing about your preferred EOL medical care		
	Odds ratio	95% CI	Pr(>|z|)	Odds ratio	95% CI	Pr(>|z|)	Odds ratio	95% CI	Pr(>|z|)	Odds ratio	95% CI	Pr(>|z|)
Age	**1.04**	**1.02; 1.07**	**0.001**	1.01	0.98; 1.03	0.491	1.00	0.97; 1.02	0.764	1.00	0.97; 1.02	0.930
HCP	0.54	0.17; 1.50	0.266	0.58	0.15; 1.84	0.385	0.76	0.19; 2.53	0.677	0.58	0.12; 1.99	0.422
Religion												
Buddhism	ref	ref	ref	ref	ref	ref	ref	ref	ref	ref	ref	ref
Christianity	1.11	0.48; 2.55	0.813	1.87	0.62; 5.99	0.273	1.27	0.39; 4.25	0.685	1.16	0.40; 3.38	0.777
No religion	0.61	0.22; 1.54	0.305	2.82	0.96; 8.98	0.065	1.83	0.56; 6.14	0.313	1.41	0.48; 4.11	0.523
Others	0.58	0.08; 2.52	0.518	2.49	0.46; 11.20	0.247	2.01	0.38; 8.93	0.370	1.07	0.15; 4.90	0.939
Gender												
Male	ref	ref	ref	ref	ref	ref	ref	ref	ref	ref	ref	ref
Female	0.76	0.36; 1.66	0.485	**7.33**	**2.07; 46.73**	**0.008**	3.57	1.15; 15.68	0.048	2.10	0.81; 6.54	0.154
Serious illness experience	1.73	0.83–3.77	0.153	2.14	0.92; 5.34	0.087	**3.65**	**1.36; 11.61**	**0.016**	1.50	0.65; 3.62	0.354
Heard of ACP	1.58	0.73; 3.52	0.253	1.02	0.41; 2.55	0.960	0.86	0.32; 2.29	0.762	0.70	0.28; 1.71	0.440

Note: Confounders adjusted for multivariate regression were heard of ACP, previous experience with serious illness, age, gender, and religion

## Discussion

We found that prior experience with serious illness, age and female gender, were significantly and independently associated with positive ACP documentation attitudes. Subgroup analyses revealed that those aged 20–39 were less likely to speak to their HCP about or sign official papers regarding EOL care. We also found that being a healthcare professional does not necessitate better or worse attitudes. We postulated several underlying reasons for the above findings.

Prior experience with serious illness was a significant predictor of readiness to discuss EOL care with a healthcare professional. Having a prior encounter with serious illness experienced by oneself or by a loved one, may confer first-hand experience of the difficulty faced by family members in making important medical decisions for their loved ones [25]. By having an ACP, patients could guide their medical decision-making when they become incapacitated and those with prior experience with serious illness may be more inclined to have positive attitudes towards it. Amjad et al. [26] and Carr et al. [27] explained that experiencing the death of a loved one who has made their EOL wishes known, increased the likelihood of ACP discussion and documentation. Furthermore, the COVID-19 pandemic may have increased the incidence of prior serious illness experiences amongst the public, in turn, affecting ACP documentation rates [28]. It is also likely that prior experience with serious illness increased exposure to the healthcare system and may increase trust in physicians to help guide patients through the ACP discussion process depending on the positiveness of the experience faced in the healthcare system [28].

Female gender was a significant predictor of readiness to discuss EOL care with their nominated healthcare spokesperson. Seifart et al. [29] found that females had significantly more EOL discussions with a psychologist and other cancer patients. Females had a higher need for information and required a more active role in decision making [29], hence increasing the likelihood of EOL discussions with their nominated healthcare spokesperson. Similarily, Faller et al. [30] reported that men had a comparatively lower requirement for information on psychological support, necessitating lesser EOL discussions. Another possible explanation could be that mothers were more concerned of causing EOL burden to their children [31]. Park et al. [31] reported in a cohort of mothers with advanced cancer that 90% were worried about the potential strain on their children at the EOL. Therefore, they are likely to be more willing to discuss their EOL care goals with their nominated healthcare spokesperson.

Age was a significant predictor of readiness to sign papers to appoint a nominated healthcare spokesperson. Moorman et al. [32] found that older adults were more likely to have discussed EOL treatment preferences and Pollack et al. [33] established that older adults were more likely to have filed for an advance directive. Furthermore, older adults were at increased risk of experiencing severe symptoms associated with COVID-19 infection and were more likely to die [34]. Potentially, the COVID-19 pandemic may have disproportionately affected ACP documentation attitudes within the older cohort [35]. Interestingly, our analysis revealed that age was not a significant predictor of readiness to discuss EOL care with their nominated healthcare spokesperson. Due to an underlying assumption by older adults that their care preferences were already known by their loved ones [36], it may not be intuitive to put their wishes down onto writing in the form of official documentation. As noted by Malcomson et al. [36], older adults may think that loved ones have already understood their wishes even with the absence of official documentation, discouraging them to initiate further ACP discussions with their nominated healthcare spokesperson. In addition, patients may avoid engaging their family members in ACP discussions for fear of possible conflicts and unrest that could negatively affect family dynamics [37]. Interestingly, subgroup analyses revealed that those aged 20–39 were less likely to speak to their healthcare professional about or sign official papers regarding EOL care. Younger adults were generally at a lower risk for life-threatening illness and may feel that ACP is less pertinent to them [38]. In addition, younger adults were still in the process of psychological development and identity transitions [39–41], and thus may be less likely to commit to a plan of action regarding their EOL care.

We found that being a healthcare professional does not necessarily imbue positive nor negative attitudes towards ACP. However, our results should be interpreted with prudence due to the small healthcare professional sample size. More focused information and training regarding ACP administration engenders positive attitudes towards ACP [42]. Chan et al. [42] found a significant association between training experience and positive ACP attitudes in a cohort of 250 healthcare professionals in Hong Kong. Further training of healthcare professionals in ACP may therefore improve willingness of administration and recommendation, translating into higher ACP uptake rates. It should be noted that healthcare professionals may have responded to certain items based on both their personal experiences being in contact with the healthcare system, and as practitioners. We recommend future studies to investigate the differences between the attitudes of healthcare professional and non- healthcare professionals towards ACP.

Strategies focusing on age, gender, and prior serious illness could increase ACP uptake amongst the population. Efforts should not only be centred on promoting the merits of ACP, but also to provide easier access to resources [43] and value-adding to the ACP process [44]. Access may be increased through online directories to clinics, hospitals and facilities employing ACP facilitators, by increasing the number of institutions offering ACP planning, and by offering teleconsultations for ACP planning. Video decision aids [45] may relieve confusion regarding various components of EOL care during the planning process. Among younger adults which are likely to not have prior serious illness experience, advocacy must emphasise the unpredictability of serious illness, even at a younger age [46]. Additionally, it should be highlighted that an ACP can be edited as priorities and values evolve with time. Further studies evaluating the reasons for age or serious illness experience increasing ACP readiness may provide more insight into formulating outreach campaigns for this group.

We also recommend palliative care organisations globally to consider adopting PHA’s approach to outreach by conducting exhibitions. The PHA exhibition comprised two sections. Firstly, an interactive, informational section comprising various boards and disseminated brochures educating on palliative care and ACP. Secondly, were partner booths, manned by representatives from Singapore Hospice Council and the Agency for Integrated Care. The informational section aimed to begin the contemplation process for ACP, where participants would be promptly guided to partner booths. Representatives would deepen understanding regarding ACP and provide resources enabling the start of the ACP process. Further collaboration between organisations should be considered to value-add towards ACP-related advocacy campaigns.

### Strengths and weaknesses of the study

The primary contribution of our paper was the collection and analysis of a standardised data set pertaining to ACP documentation attitudes in relation to demographic factors, post COVID-19 in an urbanised, multi-ethnic southeast Asian nation. A prior survey in Singapore [21], has examined associations between sociodemographic factors and ACP awareness. However, our study investigates the associations between sociodemographic factors and readiness to undertake ACP discussions and documentation, providing additional insight. Our paper had several limitations. Responses from the ACP Roadshow and PHA exhibition attendees were obtained via convenience sampling during the exhibition period and may not be fully representative of the Singaporean population. In addition, non-response bias may have arisen due to event attendees declining being surveyed. Participants who did not respond may have systematically differed in terms of demographics or documentation attitudes, ultimately resulting in possible underrepresentation of this section of the population in the results. Furthermore, the cross-sectional design of this study was liable to sampling bias and does not allow the determination of a causal relationship. Additionally, our findings may be country-specific and influenced by the Singaporean healthcare system, health legislation, and cultural context. However, the associations we drew still provided insight into the relationship between age and serious illness experience, and ACP documentation attitudes.

## Conclusion

A patient-centric approach with consideration of the influence of age and prior serious illness experience on ACP documentation attitudes may be helpful to improve advocacy by tailoring information and outreach campaigns to socio-demographic groups. Future larger cohort studies are still warranted for implementation of effective strategies to improve ACP documentation attitudes.

## Electronic supplementary material

Below is the link to the electronic supplementary material.

Supplementary Material 1

## Data Availability

Protocol, template data collection forms, data extracted from included studies, data used for analyses, and analytic code available from the corresponding author on reasonable request.
