# Correction: A cross-sectional study on advance care planning documentation attitudes during national advance care planning week in a South-East Asian country

**DOI:** 10.1186/s12904-024-01593-2

**Published:** 2024-11-22

**Authors:** Chen Ee Low, Sounak Rana, Chun En Yau, Sheryl Yen Pin Tan, Jing Ni Ng, Noreen Chan, Mervyn Jun Rui Lim

**Affiliations:** 1 Yong Loo Lin School of Medicine, National University of Singapore, Singapore, Republic of Singapore; 2 Division of Palliative Care, National University Cancer Institute, Singapore, Republic of Singapore; 3 Division of Neurosurgery, University Surgical Centre, National University Hospital, Level 8, National University Health Systems Tower Block, 1E Kent Ridge Rd, Singapore, 119228 Republic of Singapore

**Correction: BMC Palliat Care 23**,** 244 (2024).**

10.1186/s12904-024-01505-4.

Following publication of the original article [1], the authors reported that their prior request for change of the authorship was not implemented in the published article. The list of Authors, Acknowledgements, Author contribution and Funding sections should be updated as below.

**Old Author list**.

Chen Ee Low, Sounak Rana, Chun En Yau, Sheryl Yen Pin Tan, Jing Ni Ng, **Chung Min Ru**,** Kit Soh**, Noreen Chan, **Raymond Han Lip Ng** and Mervyn Jun Rui Lim.

**New Author list**.

Chen Ee Low, Sounak Rana, Chun En Yau, Sheryl Yen Pin Tan, Jing Ni Ng, Noreen Chan and Mervyn Jun Rui Lim.

**Old Authors Contribution**.

LCE and SR drafted the work. LCE, SR, YCE, ST, NJN, **CMR and KS** collected the data. LCE analysed the data. LCE, ML, NC **and RN** designed, reviewed, and substantively revised the work.

**New Author contributions**.

LCE and SR drafted the work. LCE, SR, YCE, ST, NJN collected the data. LCE analysed the data. LCE, ML, and NC designed, reviewed, and substantively revised the work.

**Old Acknowledgements**.

We acknowledge the PHA committee members and ACP Roadshow personnel.

**New Acknowledgements**.

We acknowledge the PHA committee members and ACP Roadshow personnel, **the Agency for Integrated Care**,** and Dr Raymond Ng for their support and advice for this study.**

Old Funding.

This research received no specific grant from any funding agency in the public, commercial, or not-for-profit sectors.

New Funding.

This work was supported by the Singapore Ministry of Heath’s National Medical Research Council Grants, grant number NMRC/CG1/009/2022-NUH and CareEco21-0030.

These has been corrected above and the original article has been updated.
