# Supplementary material for: A cross-sectional study on advance care planning documentation attitudes during national advance care planning week in a South-East Asian country
Source: BMC Palliat Care. 2024 Oct 17;23:244. doi: 10.1186/s12904-024-01505-4 (PMC11484203; doi:10.1186/s12904-024-01505-4)
Supplement: Supplementary file 1 — Supplementary Material 1 [file 12904_2024_1505_MOESM1_ESM.docx]

Supplementary Table 1: Subgroup logistic regression analysis of the association between the four markers of ACP Documentation Attitudes and general characteristics of HCP

Supplementary Table 2: Subgroup logistic regression analysis of the association between the four markers of ACP Documentation Attitudes and general characteristics of non-HCP

Supplementary Table 3: Subgroup logistic regression analysis of the association between the four markers of ACP Documentation Attitudes and age as a categorical variable

Supplementary Questionnaire 1: AIC Roadshow Questionnaire 2021
Supplementary Questionnaire 2: PHA Exhibition Questionnaire 2022

| HCP | **Ready to sign official papers naming a nominated healthcare spokesperson** | | | **Ready to speak with your nominated healthcare spokesperson about your preferred EOL medical care** | | | **Ready to speak with your healthcare professional about your preferred EOL medical care** | | | **Ready to sign official papers putting your wishes in writing about your preferred EOL medical care** | | |
| --- | --- | --- | --- | --- | --- | --- | --- | --- | --- | --- | --- | --- |
|  | Odds ratio | 95% CI | Pr(>\|z\|) | Odds ratio | 95% CI | Pr(>\|z\|) | Odds ratio | 95% CI | Pr(>\|z\|) | Odds ratio | 95% CI | Pr(>\|z\|) |
| **Age** | 1.01 | 0.94; 1.08 | 0.795 | 0.99 | 0.91; 1.05 | 0.777 | 1.00 | 0.91; 1.08 | 0.984 | 0.99 | 0.89; 1.07 | 0.778 |
| **Religion** |  | | | | | | | | | | | |
| Buddhism | ref | ref | ref | ref | ref | ref | ref | ref | ref | ref | ref | ref |
| Christianity | N/A | N/A | N/A | 1.29 | 0.09; 32.58 | 0.854 | 2.32 | 0.19; 70.86 | 0.542 | 1.02 | 0.07; 26.49 | 0.986 |
| No religion | 0.93 | 0.03; 17.82 | 0.960 | 1.30 | 0.04; 44.34 | 0.873 | N/A | N/A | N/A | N/A | N/A | N/A |
| Others | 1.14 | 0.04; 22.36 | 0.929 | N/A | N/A | N/A | N/A | N/A | N/A | N/A | N/A | N/A |
| **Gender** |  | | | | | | | | | | | |
| Female | ref | ref | ref | ref | ref | ref | ref | ref | ref | ref | ref | ref |
| Male | 1.32 | 0.11; 17.04 | 0.821 | N/A | N/A | N/A | N/A | N/A | N/A | N/A | N/A | N/A |
| **Serious illness experience** | N/A | N/A | N/A | 2.56 | 0.24; 62.05 | 0.52 | 1.22 | 0.08; 32.54 | 0.885 | 0.92 | 0.06; 24.63 | 0.950 |
| **Heard of ACP** | N/A | N/A | N/A | N/A | N/A | N/A | 0.15 | 0.00; 5.29 | 0.262 | N/A | N/A | N/A |

Supplementary Table 1: Subgroup logistic regression analysis of the association between the four markers of ACP Documentation Attitudes and general characteristics of HCP

Abbreviations: Not available, N/A; Reference, ref

*N/A due to insufficient sample size

Supplementary Table 2: Subgroup logistic regression analysis of the association between the four markers of ACP Documentation Attitudes and general characteristics of non-HCP

| Non-HCP | **Ready to sign official papers naming a nominated healthcare spokesperson** | | | **Ready to speak with your nominated healthcare spokesperson about your preferred EOL medical care** | | | **Ready to speak with your healthcare professional about your preferred EOL medical care** | | | **Ready to sign official papers putting your wishes in writing about your preferred EOL medical care** | | |
| --- | --- | --- | --- | --- | --- | --- | --- | --- | --- | --- | --- | --- |
|  | Odds ratio | 95% CI | Pr(>\|z\|) | Odds ratio | 95% CI | Pr(>\|z\|) | Odds ratio | 95% CI | Pr(>\|z\|) | Odds ratio | 95% CI | Pr(>\|z\|) |
| **Age** | **1.05** | **1.02; 1.08** | **0.001** | 1.01 | 0.99; 1.04 | 0.336 | 1.00 | 0.917; 1.03 | 0.746 | 1.00 | 0.97; 1.03 | 0.988 |
| **Religion** |  | | | | | | | | | | | |
| Buddhism | ref | ref | ref | ref | ref | ref | ref | ref | ref | ref | ref | ref |
| Christianity | 1.54 | 0.62; 3.83 | 0.348 | 1.93 | 0.56; 7.08 | 0.297 | 0.85 | 0.19; 3.49 | 0.819 | 1.02 | 0.31; 3.33 | 0.967 |
| No religion | 0.65 | 0.22; 1.79 | 0.414 | 3.12 | 0.98; 11.07 | 0.061 | 2.16 | 0.63; 7.98 | 0.226 | 1.60 | 0.53; 4.95 | 0.398 |
| Others | 0.38 | 0.02; 2.45 | 0.393 | 4.27 | 0.74; 22.05 | 0.084 | 3.04 | 0.53; 15.46 | 0.185 | 1.42 | 0.19; 7.09 | 0.690 |
| **Gender** |  | | | | | | | | | | | |
| Male | ref | ref | ref | ref | ref | ref | ref | ref | ref | ref | ref | ref |
| Female | 0.79 | 0.35; 1.85 | 0.581 | **6.90** | **1.90; 44.63** | **0.012** | 3.24 | 1.00; 14.70 | 0.077 | 1.89 | 0.71; 5.98 | 0.235 |
| **Serious illness experience** | 1.45 | 0.66; 3.27 | 0.362 | 2.07 | 0.83; 5.53 | 0.130 | **4.25** | **1.42; 15.89** | **0.016** | 1.52 | 0.62; 3.90 | 0.363 |
| **Heard of ACP** | 1.44 | 0.65; 3.25 | 0.374 | 0.93 | 0.36; 2.38 | 0.880 | 1.06 | 0.38; 2.99 | 0.911 | 0.65 | 0.25; 1.62 | 0.362 |

Abbreviations: Not available, N/A; Reference, ref

*N/A due to insufficient sample size

Supplementary Table 3: Subgroup logistic regression analysis of the association between the four markers of ACP Documentation Attitudes and age as a categorical variable

| Overall | **Ready to sign official papers naming a nominated healthcare spokesperson** | | | **Ready to speak with your nominated healthcare spokesperson about your preferred EOL medical care** | | | **Ready to speak with your healthcare professional about your preferred EOL medical care** | | | **Ready to sign official papers putting your wishes in writing about your preferred EOL medical care** | | |
| --- | --- | --- | --- | --- | --- | --- | --- | --- | --- | --- | --- | --- |
|  | Odds ratio | 95% CI | Pr(>\|z\|) | Odds ratio | 95% CI | Pr(>\|z\|) | Odds ratio | 95% CI | Pr(>\|z\|) | Odds ratio | 95% CI | Pr(>\|z\|) |
| **Age** |  | | | | | | | | | | | |
| **>60** | **ref** | **ref** | **ref** | **ref** | **ref** | **ref** | **ref** | **ref** | **ref** | **ref** | **ref** | **ref** |
| **<20** | 0.29 | 0.01; 1.80 | 0.264 | N/A | N/A | N/A | N/A | N/A | N/A | N/A | N/A | N/A |
| **20-39** | 0.90 | 0.37; 2.27 | 0.826 | 0.98 | 0.36; 2.81 | 0.965 | **0.24** | **0.05; 0.91** | **0.047** | **0.28** | **0.07; 0.94** | **0.046** |
| **40-59** | 0.59 | 0.23; 1.51 | 0.266 | 0.85 | 0.31; 2.46 | 0.751 | 1.14 | 0.44; 3.21 | 0.792 | 1.07 | 0.43; 2.84 | 0.892 |
| **Religion** |  | | | | | | | | | | | |
| Buddhism | ref | ref | ref | ref | ref | ref | ref | ref | ref | ref | ref | ref |
| Christianity | 1.11 | 0.48; 2.55 | 0.813 | 1.87 | 0.62; 5.99 | 0.273 | 1.27 | 0.39; 4.25 | 0.685 | 1.16 | 0.40; 3.38 | 0.777 |
| No religion | 0.61 | 0.22; 1.54 | 0.305 | 2.82 | 0.96; 8.98 | 0.065 | 1.83 | 0.56; 6.14 | 0.313 | 1.41 | 0.48; 4.11 | 0.523 |
| Others | 0.58 | 0.08; 2.52 | 0.518 | 2.49 | 0.46; 11.20 | 0.247 | 2.01 | 0.38; 8.93 | 0.370 | 1.07 | 0.15; 4.90 | 0.939 |
| **Gender** |  | | | | | | | | | | | |
| Male | ref | ref | ref | ref | ref | ref | ref | ref | ref | ref | ref | ref |
| Female | 0.76 | 0.36; 1.66 | 0.485 | 7.33 | 2.07; 46.73 | 0.008 | 3.57 | 1.15; 15.68 | 0.048 | 2.10 | 0.81; 6.54 | 0.154 |
| **Serious illness experience** | 1.73 | 0.83-3.77 | 0.153 | 2.14 | 0.92; 5.34 | 0.087 | 3.65 | 1.36; 11.61 | 0.016 | 1.50 | 0.65; 3.62 | 0.354 |
| **Heard of ACP** | 1.58 | 0.73; 3.52 | 0.253 | 1.02 | 0.41; 2.55 | 0.960 | 0.86 | 0.32; 2.29 | 0.762 | 0.70 | 0.28; 1.71 | 0.440 |

Abbreviations: Not available, N/A; Reference, ref

*N/A due to insufficient sample size

Supplementary Questionnaire 1: AIC Roadshow Questionnaire 2021
**Introduction**

Thank you for taking the time to participate in this survey. Before doing so, kindly read the consent form to ensure that you understand the study and what is involved.

☐ I have read and understood the consent form and consent to participation in this study.

*[Participant information sheet will be attached at the start of the survey.]*

# **Section A: Socio-demographics**

This section contains several statements related to your socio-demographics. Please read each statement carefully, and answer the questions.

1. What is your gender?

☐Male

☐Female

2. What is your age? (in years): _______

3. What is your race?

☐Chinese

☐Malay

☐Indian

☐Others (please specify):___________

4. What is your religion?

☐Buddhism

☐Christianity

☐Hinduism

☐Islam

☐No Religion

☐Others (please specify):____________

5. What is your nationality?

☐Singaporean

☐Permanent Resident

☐Others (please specify):___________

6. What is your marital status?

☐Single

☐Married

☐Divorced

☐Widowed

☐Others (please specify):___________

7. Are you a healthcare worker? Healthcare worker: someone who works in the healthcare sector (i.e. doctor, nurse, allied health professional, etc)

☐Yes

☐No

8. Have you ever had experience with serious illness (for example, being admitted into the Intensive Care Unit), either yourself, or a family member or friend?

☐Yes

☐No

9. Have you thought about what would be important to you if you were near the end of life?

☐Yes

☐No

10.Have you heard of Advance Care Planning (ACP)?

☐Yes

☐No

9a. If yes, where did you first hear about ACP?

☐National media (e.g. newspapers, news reports, magazines, etc)

☐Family and friends

☐Activities/talks at National ACP Week 2022

☐Others (please specify):____________

This section contains several statements related to your personal engagement with ACP. Please read each statement carefully, and select the option that best describes yourself. There are no correct or wrong answers, so please answer the questions truthfully.

**This question asks about Nominated Healthcare Spokespersons. A Nominated Healthcare Spokesperson (NHS) is a family member or friend who can speak for you if you were to become too sick to speak for yourself.**

|  | I have never thought about it | I have thought about it, but I am not ready to do it | I am thinking about doing it in the next 6 months | I am definitely planning to do it in the next 30 days | I have already done it |
| --- | --- | --- | --- | --- | --- |
| 11. How ready are you to SIGN OFFICIAL PAPERS naming a person or group of people to be your spokesperson or to make decisions for you? |  |  |  |  |  |

**The following questions are about specific medical treatments (for example, performing emergency resuscitation, being put on a breathing tube, etc) that people may or may never want if they were near the end of life. Please give us your honest opinions to the following questions about medical treatments.** *There are no right or wrong answers.*

|  | I have never thought about it | I have thought about it, but I am not ready to do it | I am thinking about doing it in the next 6 months | I am definitely planning to do it in the next 30 days | I have already done it |
| --- | --- | --- | --- | --- | --- |
| 12. How ready are you to talk with your NHS about the kind of medical care you would want if you were near the end of life? |  |  |  |  |  |
| 13. How ready are you to talk with your HEALTHCARE PROFESSIONAL about the kind of medical care you would want if you were near the end of life? |  |  |  |  |  |
| 14. How ready are you to SIGN OFFICIAL PAPERS putting your wishes in writing about the kind of medical care you would want if you were near the end of life? |  |  |  |  |  |

The following 2 statements are related to different attitudes toward death. Read each statement carefully, and please select between “Strongly Disagree (1)” to “Strongly Agree (7)” to indicate your response. There are no correct or wrong answers, so please answer the questions truthfully.

|  | Strongly Disagree (1) | Disagree (2) | Moderately Disagree (3) | Neutral (4) | Moderately Agree (5) | Agree (6) | Strongly Agree (7) |
| --- | --- | --- | --- | --- | --- | --- | --- |
| 15. Thinking about my own death makes me anxious. |  |  |  |  |  |  |  |
| 16. I avoid death thoughts at all costs. |  |  |  |  |  |  |  |

**PHA Post-exhibition survey**

1. What is your gender?

☐Male

☐Female

☐Others, please specify:

1. What is your age (years)?
2. What is your highest level of education?

☐Primary

☐Secondary

☐Tertiary

☐University

☐Post-Graduate

☐Did not receive any education

1. What is your religion?

☐Buddhism

☐Christianity

☐Hinduism

☐Catholicism

☐No religion

☐Others, please specify:

1. What is your monthly income (in SGD)?

☐$0

☐<$1000

☐$1000 t0 $2999

☐$3000 to $4999

☐$5000 to $6999

☐$7000 to $899

☐>$9000

1. What is your nationality?

☐Singaporean

☐Permanent Resident

☐Others, please specify:

1. What is your marital status?

☐Single

☐Married

☐Divorced

☐Widowed

☐Others, please specify:

1. Are you a healthcare worker?

Healthcare worker: Someone who works in the healthcare sector (E.g. Doctor, Nurse, Allied health professional).

☐No

☐Yes

1. Have you ever had experience with serious illness either yourself, or a family member or friend?

For example, being admitted into the Intensive Care Unit

☐No

☐Yes

1. Have you thought about what would be important to you if you were near the end of life, prior to the exhibition?

☐No

☐Yes

**Engagement with Advance Care Planning (ACP)**
This section contains several statements related to your personal engagement with ACP. Please read each statement carefully and select the option that best describes yourself. There are no correct or wrong answers, so please answer the questions truthfully.

1. How ready are you to SIGN OFFICIAL PAPERS naming a person or group of people to be your spokesperson or to make decisions for you?

This question asks about Nominated Healthcare Spokespersons. A Nominated Healthcare Spokesperson (NHS) is a family member or friend who can speak for you if you were to become too sick to speak for yourself.

☐I have never thought about it

☐I have thought about it, but I am not ready to do it

☐I am thinking about doing it in the next 6 months

☐I am definitely planning to do it in the next 30 days

☐I have already done it

1. The following questions are about specific medical treatments (E,g performing emergency resuscitation, being put on a breathing tube) that people may or may never want if they were near the end of life.

There are no correct or wrong answers, so please answer the questions truthfully.

- 1. How ready are you to talk with your Nominated Healthcare Spokesperson (NHS) about the kind of medical care you would want if you were near the end of life?

☐I have never thought about it

☐I have thought about it, but I am not ready to do it

☐I am thinking about doing it in the next 6 months

☐I am definitely planning to do it in the next 30 days

☐I have already done it

- 1. How ready are you to talk with your HEALTHCARE PROFESSIONAL about the kind of medical care you would want if you were near the end of life?

☐I have never thought about it

☐I have thought about it, but I am not ready to do it

☐I am thinking about doing it in the next 6 months

☐I am definitely planning to do it in the next 30 days

☐I have already done it

- 1. How ready are you to SIGN OFFICIAL PAPERS putting your wishes in writing about the kind of medical care you would want if you were near the end of life?

☐I have never thought about it

☐I have thought about it, but I am not ready to do it

☐I am thinking about doing it in the next 6 months

☐I am definitely planning to do it in the next 30 days

☐I have already done it

1. The following statements are related to different attitudes toward death.

Read each statement carefully, and please select between “Strongly Disagree (1)” to “Strongly Agree (7)” to indicate your response. There are no correct or wrong answers, so please answer the questions truthfully.

- 1. Thinking about my own death makes me anxious.

☐Strongly disagree (1)

☐ Disagree (2)

☐Moderately disagree (3)

☐Neutral (4)

☐Moderately agree (5)

☐Agree (6)

☐Strongly agree (7)

- 1. I avoid death thoughts at all costs.

☐Strongly disagree (1)

☐Disagree (2)

☐Moderately disagree (3)

☐Neutral (4)

☐Moderately agree (5)

☐Agree (6)

☐Strongly agree (7)

**Knowledge & Perceptions about Palliative Care**

This section contains several questions regarding your attitude towards palliative care, prior to the exhibition and after.

1. BEFORE the exhibition, my level of familiarity towards the issues surrounding palliative care is

☐Completely unfamiliar (1)

☐Unfamiliar (2)

☐Neutral (3)

☐Familiar (4)

☐Very familiar (5)

1. AFTER the exhibition, my level of familiarity towards the issues surrounding palliative care is

☐Completely unfamiliar (1)

☐Unfamiliar (2)

☐Neutral (3)

☐Familiar (4)

☐Very familiar (5)

1. BEFORE the exhibition, my understanding towards the concerns of palliative care patients is

☐Completely do not understand (1)

☐Do not understand (2)

☐Neutral (3)

☐Understand (4)

☐Completely understand (5)

1. AFTER the exhibition, my understanding towards the concerns of palliative care patients is

☐Completely do not understand (1)

☐Do not understand (2)

☐Neutral (3)

☐Understand (4)

☐Completely understand (5)

1. BEFORE the exhibition, my comfort level when talking about death is

☐Very uncomfortable (1)

☐Uncomfortable (2)

☐Neutral (3)

☐Comfortable (4)

☐Very comfortable (5)

1. AFTER the exhibition, my comfort level when talking about death is

☐Very uncomfortable (1)

☐Uncomfortable (2)

☐Neutral (3)

☐Comfortable (4)

☐Very comfortable (5)

1. BEFORE the exhibition, when I think of palliative care, I automatically think of death.

☐Strongly disagree (1)

☐Disagree (2)

☐Neutral (3)

☐Agree (4)

☐Strongly agree (5)

1. AFTER the exhibition, when I think of palliative care, I automatically think of death.

☐Strongly disagree (1)

☐Disagree (2)

☐Neutral (3)

☐Agree (4)

☐Strongly agree (5)

1. BEFORE the exhibition, I have heard of Advance Care Planning.

☐No

☐Yes

1. AFTER the exhibition, I understand the importance of Advance Care Planning.

☐Strongly disagree (1)

☐Disagree (2)

☐Neutral (3)

☐Agree (4)

☐Strongly agree (5)

1. AFTER the exhibition, I am likely to start Advance Care Planning.

☐Strongly disagree (1)

☐Disagree (2)

☐Neutral (3)

☐Agree (4)

☐Strongly agree (5)

1. When thinking about end-of-life, how important are the following factors to you?

Among the 4 factors, rank them from the MOST important to the LEAST important Companionship (family/friends/loved ones)

Relief of suffering

Life completion activities (To fulfill final wishes)

Having family cared for after death

☐Most important (1)

☐Important (2)

☐Not important (3)

☐Least important (4)

1. When thinking about the end-of-life, besides the factors mentioned above, what other important factors would you consider?

**Exhibition Feedback**

This section asks several questions to gauge your response to the exhibition, and how much it may have benefitted you

1. I found the exhibition informative.

☐Strongly disagree (1)

☐Disagree (2)

☐Neutral (3)

☐Agree (4)

☐Strongly agree (5)

1. Which exhibition activities did you participate in?

☐Postcard writing

☐Quiz Game – How Well Do I Know Palliative Care?

☐Conversation starters.- How Well Do I Know Your Loved Ones?

☐Snakes and Ladders – What Choices Will You Make?

1. How useful were the activities in helping to improve your understanding of palliative care?

Among the 4 activities, rank them from the MOST useful to the LEAST useful.

Postcard writing

Quiz Game - How Well Do I Know Palliative Care?

Conversation Starters - How Well Do You Know Your Loved Ones?

Snakes and Ladders - What Choices Will You Make?

☐Most useful (1)

☐Useful (2)

☐Not useful (3)

☐Least useful (4)

1. How much did you enjoy the activities?
   1. Postcard writing

☐Thoroughly did not enjoy (1)

☐Did not enjoy (2)

☐Neutral (3)

☐Enjoy (4)

☐ Thoroughly enjoy (5)

- 1. Quiz Game - How Well Do I Know Palliative Care

☐Thoroughly did not enjoy (1)

☐Did not enjoy (2)

☐Neutral (3)

☐Enjoy (4)

☐ Thoroughly enjoy (5)

- 1. Conversation Starters - How Well Do You Know Your Loved Ones?

☐Thoroughly did not enjoy (1)

☐Did not enjoy (2)

☐Neutral (3)

☐Enjoy (4)

☐ Thoroughly enjoy (5)

- 1. Snakes and Ladders - What Choices Will You Make?

☐Thoroughly did not enjoy (1)

☐Did not enjoy (2)

☐Neutral (3)

☐Enjoy (4)

☐ Thoroughly enjoy (5)

1. I found the booths well-designed.

☐Strongly disagree (1)

☐Disagree (2)

☐Neutral (3)

☐Agree (4)

☐Strongly agree (5)

1. We were able to answer all of your questions regarding palliative care.

☐Strongly disagree (1)

☐Disagree (2)

☐Neutral (3)

☐Agree (4)

☐Strongly agree (5)

1. I am likely to attend similar events in the future

☐Strongly disagree (1)

☐Disagree (2)

☐Neutral (3)

☐Agree (4)

☐Strongly agree (5)

1. I will recommend others to attend this exhibition.

☐Strongly disagree (1)

☐Disagree (2)

☐Neutral (3)

☐Agree (4)

☐Strongly agree (5)

1. What is your biggest takeaway from this exhibition?
2. How can we improve?

Any feedback is highly appreciated.

Supplementary Questionnaire 2: PHA Questionnaire 20212

By filling this form, you agree to participate in the Project Happy Apples (PHA)'s Post-Exhibition Survey.

Your participation in this study is voluntary. You may stop participating in this study at any time. Your decision not to take part in this study or to stop your participation will not affect your medical care or any benefits to which you are entitled. If you decide to stop taking part in this study, you should tell the Principal Investigator or any staff members. If you withdraw from the study, there will be no consequences However, the data that have been collected until the time of your withdrawal will be kept and analysed. The reason is to enable a complete and comprehensive evaluation of the study.

Your doctor, the Investigator and/or the Sponsor of this study may stop your participation in the study at any time if they decide that it is in your best interests. They may also do this if you do not follow instructions required to complete the study adequately. If you have other medical problems or side effects, the doctor and/or nurse will decide if you may continue in the research study.

In the event of any new information becoming available that may be relevant to your willingness to continue in this study, you (or your legally acceptable representative, if relevant) will be informed in a timely manner by the Principal Investigator or his/her representative.

There will be no circumstances under which the participant will be contacted for further consent as this is a once-off health service evaluative survey.

Compliance to Personal Data Protection Act (PDPA): By indicating your consent to provide your personal data in this form, you agree to receive publicity materials, updates and important announcements by email and phone. I hereby declare all information I have provided as accurate, and understand that my information may be passed to relevant committee members for the purposes of contacting me and event organisation. All personal information will be kept confidential and used for the purpose(s) stated. I hereby give my consent to the survey-taker to collect my personal information for disseminating more information about this event and, from time to time, be called, sent SMS-es, text messages or emails on more details pertaining to, or related to, this event. Should you wish to withdraw your consent for us to contact you for the purposes stated above, please notify us in writing to @rouyii. We will then remove your personal information from our database. Please allow at least 7 business days for your withdrawal of consent to take effect. Should you have any other queries, please contact the above.

* Required

1. What is your gender? *

*Mark only one oval.*

Male Female

Other:

1. What is your age (in years)? *
2. What is your highest level of education? *

*Mark only one oval.*

Primary Secondary Tertiary University Post Graduate

1. What is your religion? *

*Mark only one oval.*

Buddhism Christianity Hinduism Islam Catholicism No religion

Other:

1. What is your monthly income? *

*Mark only one oval.*

$0

< $1000

$1000-2999

$3000-4999

$5000-6999

$7000-8999

>$9000

1. What is your nationality? *

*Mark only one oval.*

Singaporean Permanent Resident

Other:

1. What is your marital status? *

*Mark only one oval.*

Single Married Divorced Widowed

Other:

1. Are you a healthcare worker? Healthcare worker: someone who works in the * healthcare sector (E.g. Doctor, Nurse, Allied health professional)

*Mark only one oval.*

Yes No

1. Have you ever had experience with serious illness (for example, being * admitted into the Intensive Care Unit), either yourself, or a family member or friend?

*Mark only one oval.*

Yes No

1. Have you thought about what would be important to you if you were near * the end of life, prior to the exhibition?

*Mark only one oval.*

Yes No

Engagement with ACP

This section contains several statements related to your personal engagement with ACP. Please read each statement carefully and select the option that best describes yourself. There are no correct or wrong answers, so please answer the questions truthfully.

1. This question asks about Nominated Healthcare Spokespersons. A Nominated * Healthcare Spokesperson (NHS) is a family member or friend who can speak

for you if you were to become too sick to speak for yourself.

*Check all that apply.*

I have never thought about it

I have thought about it, but I am not ready to do it

I am thinking about doing it in the next 6 months

I am definitely planning to do it in the next 30 days

I have already done it

How ready are you to SIGN OFFICIAL PAPERS

naming a person or group of people to be your spokesperson or to make decisions for you?

1. The following questions are about specific medical treatments (E,g * performing emergency resuscitation, being put on a breathing tube) that

people may or may never want if they were near the end of life. There are no correct or wrong answers, so please answer the questions truthfully.

*Check all that apply.*

I have never thought about it

I have thought about it, but I am not ready to do it

I am thinking about doing it in the next 6 months

I am definitely planning to do it in the next 30 days

I have already done it

How ready are you to talk with your NHS about the kind of medical care you would want if you were near the end of life?

How ready are you to talk with your HEALTHCARE PROFESSIONAL

about the kind of medical care you would want if you were near the end of life?

How ready are you to SIGN OFFICIAL

PAPERS putting your wishes in writing about the kind of medical care you would want if you were near the end of life?

1. The following 2 statements are related to different attitudes toward death. Read * each statement carefully, and please select between “Strongly Disagree (1)” to “Strongly Agree (7)” to indicate your response. There are no correct or wrong answers, so please answer the questions truthfully.

*Check all that apply.*

Strongly Disagree (1)

Disagree (2)

Moderately Disagree (3)

Neutral (4)

Moderately Agree (5)

Agree (6)

Strongly Agree (7)

Thinking about my own death makes me anxious.

I avoid death thoughts at all costs.

Knowledge and Perceptions about palliative care

This section contains several questions regarding your attitude towards palliative care, prior to the exhibition and after.

1A) Before the exhibition, my level of familiarity towards the issues surrounding * palliative care is

*Mark only one oval.*

1 2 3 4 5

Completely unfamiliar Very familiar

1B) After the exhibition, my level of familiarity towards the issues surrounding * palliative care is

*Mark only one oval.*

1 2 3 4 5

Completely unfamiliar Very familiar

2A) Before the exhibition, my understanding towards the concerns of * palliative care patients is

*Mark only one oval.*

1 2 3 4 5

Completely do not understand Understand very well

2B) After the exhibition, my understanding towards the concerns of * palliative care patients is

*Mark only one oval.*

1 2 3 4 5

Completely do not understand Understand very well

3A) Before the exhibition, my comfort level when talking about death is *

*Mark only one oval.*

1 2 3 4 5

Not comfortable at all Very comfortable

3B) After the exhibition, my comfort level when talking about death is *

*Mark only one oval.*

1 2 3 4 5

Not comfortable at all Very comfortable

4A) Before the exhibition, when I think of palliative care, I automatically think of death. *

*Mark only one oval.*

1 2 3 4 5

Strongly agree Strongly disagree

4B) After the exhibition, when I think of palliative care, I automatically think of death. *

*Mark only one oval.*

1 2 3 4 5

Strongly agree Strongly disagree

5A) Before the exhibition, I have heard of Advanced Care Planning. *

*Mark only one oval.*

Yes No

5B) If yes, where did you first hear about ACP? *

*Mark only one oval.*

National media (e.g. newspapers, news reports, magazines)

Family and friends

Activities

Other:

5C) After the exhibition, I understand the importance of Advanced Care * Planning (ACP)

*Mark only one oval.*

1 2 3 4 5

Strongly disagree Strongly agree

5D) After the exhibition, I am likely to start Advanced Care Planning (ACP) *

*Mark only one oval.*

1 2 3 4 5

Strongly disagree Strongly agree

6A) When thinking about the end-of-life, how important are the following * factors? Rank each option according to its level of importance (1= Most important, 4=Least important)

*Check all that apply.*

1 (Most important)

2 3 4 5 (Least important)

Companionship (family/friends/loved ones)

Relief of suffering

Life completion activities (To fulfill final wishes)

Having family cared for after death

6B) When thinking about the end-of-life, besides the factors mentioned above, what * other important factors would you consider?

Exhibition feedback

This section asks several questions to gauge your response to the exhibition, and how much it may have benefitted you

1. I found the exhibition informative *

*Mark only one oval.*

1 2 3 4 5

Strongly disagree Strongly agree

1. Which exhibition activities did you participate in? *

*Check all that apply.*

Postcard writing

Quiz Game- How well do I know palliative care?

Conversation starters - How Well Do You Know Your Loved Ones? Snakes and Ladders - What Choices Will You Make?

1. On a scale of 1 to 5, how useful were the activities in helping your * understanding of palliative care (1= Most Helpful, 5=Least helpful).

*Check all that apply.*

1 (Most 2 3 4

helpful)

5

(Least helpful)

Postcard writing

Quiz Game- How well do I know palliative care?

Conversation starters - How Well Do You Know Your Loved Ones?

Snakes and Ladders - What Choices Will You Make?

1. On a scale of 1 to 5, how much did you enjoy the activities? (1=Did not * enjoy, 5=Enjoyed it very much).

*Check all that apply.*

1 (Did not enjoy)

2 3 4

5

(Enjoyed it very much)

Postcard writing

Quiz Game- How well do i know palliative care?

Conversation starters - How Well Do You Know Your Loved Ones?

Snakes and Ladders - What Choices Will You Make?

1. I found the booths well-designed *

*Mark only one oval.*

1 2 3 4 5

Strongly disagree Strongly agree

1. We were able to answer all of your questions regarding “Palliative Care” *

*Mark only one oval.*

1 2 3 4 5

No Yes

1. I am likely to attend similar events in the future. *

*Mark only one oval.*

1 2 3 4 5

Strongly disagree Strongly agree

1. I will recommend others to attend this exhibition. *

*Mark only one oval.*

1 2 3 4 5

Strongly disagree Strongly agree

1. What is your biggest takeaway from this exhibition? *
2. We would love to hear a quote from you? Please share here *
3. How can we improve? Any feedback is appreciated *
